# A bibliometric analysis of the immune system and cognitive impairment: trends from 1985 to 2024

**DOI:** 10.3389/fnagi.2025.1587575

**Published:** 2025-07-28

**Authors:** Beibei Zou, Jinxi Xiang, Muhua Zhang, Jing Huang, Chao Feng

**Affiliations:** ^1^Department of Cardiology, the Fourth Affiliated Hospital of School of Medicine, International School of Medicine, International Institutes of Medicine, Zhejiang University, Yiwu, China; ^2^Shanghai Weiyu High School, Shanghai, China; ^3^Xuhui District Central Hospital, Shanghai, China

**Keywords:** cognitive impairment, immune system, bibliometric, neuroinflammation, microglia, gut microbiota, TREM2, co-citation

## Abstract

**Background:**

Cognitive impairment is closely linked to immune system dysfunction, with increasing research interest in the underlying mechanisms and potential therapeutic targets. Bibliometric analysis provides a comprehensive approach to understanding research trends, influential contributions, and emerging topics in this interdisciplinary field.

**Methods:**

This study conducted a bibliometric analysis of publications related to the immune system and cognitive impairment from 1985 to 2024, retrieved from the Web of Science Core Collection. CiteSpace (6.4. R1), VOSviewer (1.6.20), and R-bibliometrix (R 4.3.0) were employed to analyze publication trends, co-authorship networks, keyword clustering, and co-citation patterns. Key metrics, including the H-index, G-index, and M-index, were computed to assess academic influence.

**Results:**

A total of 3,737 publications were analyzed, revealing a significant increase in research output since 2021. The United States and China emerged as leading contributors, with a robust presence of collaborative networks. Keyword and co-citation analysis identified core research themes, including neuroinflammation, microglia activation, gut microbiota, TREM2-mediated immune responses, and inflammasomes. Emerging topics such as the gut–brain axis, metabolic syndromes, and immune regulation in neurodegenerative diseases have gained prominence in recent years. Highly cited papers highlighted the role of immune dysregulation in Alzheimer’s disease, multiple sclerosis, and HIV-associated neurocognitive disorders.

**Conclusion:**

This bibliometric analysis provides a comprehensive overview of research trends in immune-related cognitive impairment. The findings indicate an increasing focus on neuroinflammatory mechanisms, immune cell interactions, and novel immunotherapeutic strategies. Future research is expected to further explore the gut–immune–brain axis and precision medicine approaches in managing cognitive disorders. These findings may facilitate early detection strategies and novel interventions targeting immune–cognitive interactions, such as gut–brain axis modulation.

## 1 Introduction

The immune system in vertebrates consists of two primary subsystems: the innate and adaptive immune systems (Mohmmad-Rezaei et al., 2021). The innate immune system responds to pathogens through germline-encoded receptors, which are expressed particularly in cell types that are not clonally distributed. Pattern recognition receptors (PRRs), such as Toll-like receptors (TLRs), recognize microbial pathogens and damage-associated molecular patterns (DAMPs)—including misfolded proteins, denatured DNA, and lipopolysaccharides (LPS)—playing a crucial role in initiating NF-κB signaling and inflammation (Barton et al., 2014). The cellular components of the innate immune system consist of dendritic cells (DCs), monocytes, macrophages (as well as microglia in the brain), and natural killer T cells. The innate immune system responds rapidly to triggers, but its response is relatively nonspecific. In contrast, the adaptive immune response can be very specific. This specificity depends on the large repertoires of antigen receptors on T and B cells (TCRs and BCRs), the cellular elements of the adaptive immune system (Clark, 2006).

Both the innate and adaptive immune responses are present in the central nervous system (CNS), although the immune niche is safeguarded as “immune privileged” in the homeostatic brain parenchyma. Various studies on neurodegenerative diseases, including Alzheimer’s disease (AD), have demonstrated the involvement of both innate and adaptive immune responses in the initiation and progression of the disease, indicating the remodeling of the immune state and the cooperation of both arms of immunity within the brain parenchyma (Amor and Woodroofe, 2014).

Recent studies have shown that high levels of circulating C-reactive protein, IL-1, IL-6, tumor necrosis factor-*α* (TNF-α), and CD4 + T-cell count might increase the risk of dementias (Gate et al., 2021; Contreras et al., 2022; Zhao et al., 2024). This new focus has been supported by epidemiological studies that link chronic inflammatory diseases (for example, diabetes, autoimmune diseases, and severe infections) to an increased risk of dementia (Gudala et al., 2013; Sipilä et al., 2021; Cooper et al., 2023). It is widely believed that in a healthy state, the blood–brain barrier (BBB) protects the CNS from peripheral neurotoxic molecules and pathogens, keeping the CNS immune privileged (Wei et al., 2024). However, aging and peripheral inflammation that arises from low-grade systemic inflammation and infections can disrupt this function (Geloso et al., 2024; Quiros-Roldan et al., 2024; Tamatta et al., 2025). A dysfunctional BBB might promote the expression of endothelial adhesion molecules and chemokines, lead to the migration of peripheral leukocytes into the CNS (Geloso et al., 2024), and then result in the activation of the central immune system and exposure of the CNS to prolonged neuroinflammation and subsequent neurodegeneration.

Cognitive impairment is a common complication of various diseases that can affect the neurological system, such as HIV infection and multiple sclerosis. An increasing number of studies have explored the potential risk factors and underlying mechanisms of cognitive impairment, including the role of the immune system. However, the pathogenesis of cognitive impairment varies significantly across conditions and remains poorly understood. This complexity underscores the need for more integrated, cross-disease investigations that incorporate both clinical and immunological perspectives. While prior bibliometric studies have mapped general trends, gaps remain in cross-disease immune mechanisms (e.g., comparing Alzheimer’s disease, HIV, and multiple sclerosis) and the longitudinal evolution of research themes. This study aims to address these gaps by analyzing 40-year trends, emerging molecular topics (e.g., TREM2 and NLRP3), and global collaboration networks to identify underexplored therapeutic targets. In this study, bibliometric methods were adopted to summarize the previous studies on the immune system and cognitive impairment. Based on this study, we aim to summarize the hotspots in this area and indicate potential directions for future research.

## 2 Materials and methods

### 2.1 Data sources and collection

**Figure 1:**
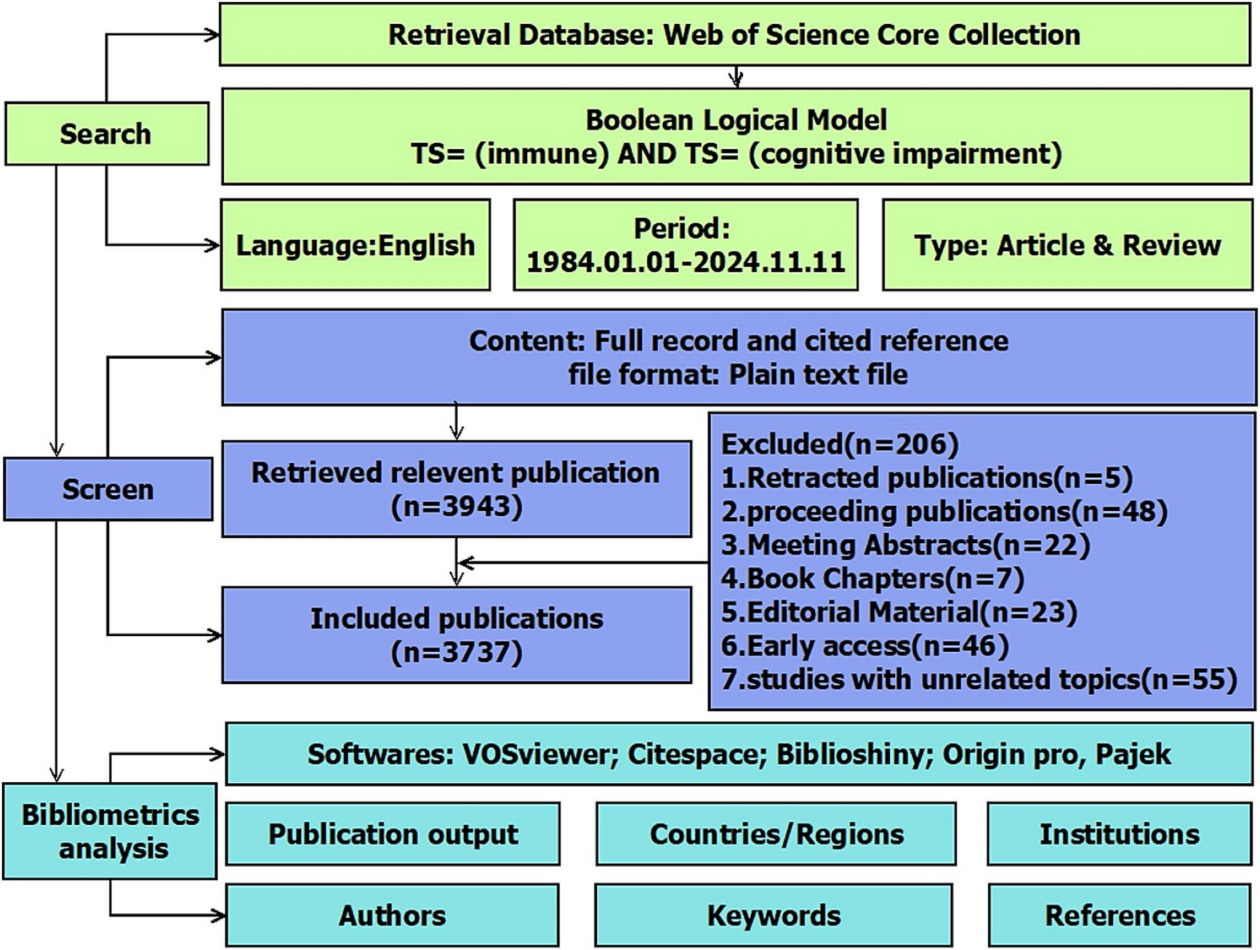
Flowchart for the selection of publications included in this study.

This study utilized the Web of Science (WOS) as its primary database. To mitigate potential errors from database updates, we promptly acquired all source data from the WOS Core Collection within a single day (November 11, 2024). We included early access and in-press articles available at that time, provided they had confirmed metadata and DOI numbers. Given concerns about redundancy and peripheral literature with “topic” retrieval, data were retrieved from the Science Citation Index Expanded web database in the WOS Core Collection. We considered the comprehensiveness, timeliness, and relevance of the sources. We used the following Boolean search string in the Web of Science Core Collection: TS = (“cognitive impairment”) AND TS = (“immune”). Synonyms and truncated terms (e.g., “neurodegeneration*”) were not included to ensure specificity. Excluded records included 5 retracted articles, 48 conference proceedings, and other non-peer-reviewed document types (Figure 1). From 1985 to 2024, a total of 3,737 publications were obtained, all of which were reviews and articles in English, downloaded under the name “XXX_download.txt” for further bibliometric visualization and analysis. The exact operational procedure is shown in Figure 1.

### 2.2 Data analysis

In the study, CiteSpace (version 6.4. R1) software, developed by Prof. Chaomei Chen, was employed for burst analysis and clustering of keywords and references. VOSviewer (version 1.6.20) was used to visually represent co-occurrence networks of countries, institutions, journals, authors, and keywords, as well as co-citation networks of authors and journals. R-bibliometrix (version R 4.3.0) and R-Studio were used to analyze detailed publication trends for countries, institutions, authors, and journals. Moreover, metrics such as the H-index, G-index, and M-index were computed for countries, journals, and authors. However, we acknowledge that these indicators may underrepresent newly emerging scholars due to citation lag. OriginPro 2024 (version 10.1.0.178) was used for visualizing publication and citation numbers. Pajek offers various layout algorithms and beautification options for VOSviewer, enhancing the clarity and presentation of network structures. In this study, it was mainly used for optimizing the layout of keyword co-occurrence networks. The document selection and analysis process is illustrated in Figure 1.

## 3 Results

### 3.1 Trend and annual count

**Figure 2:**
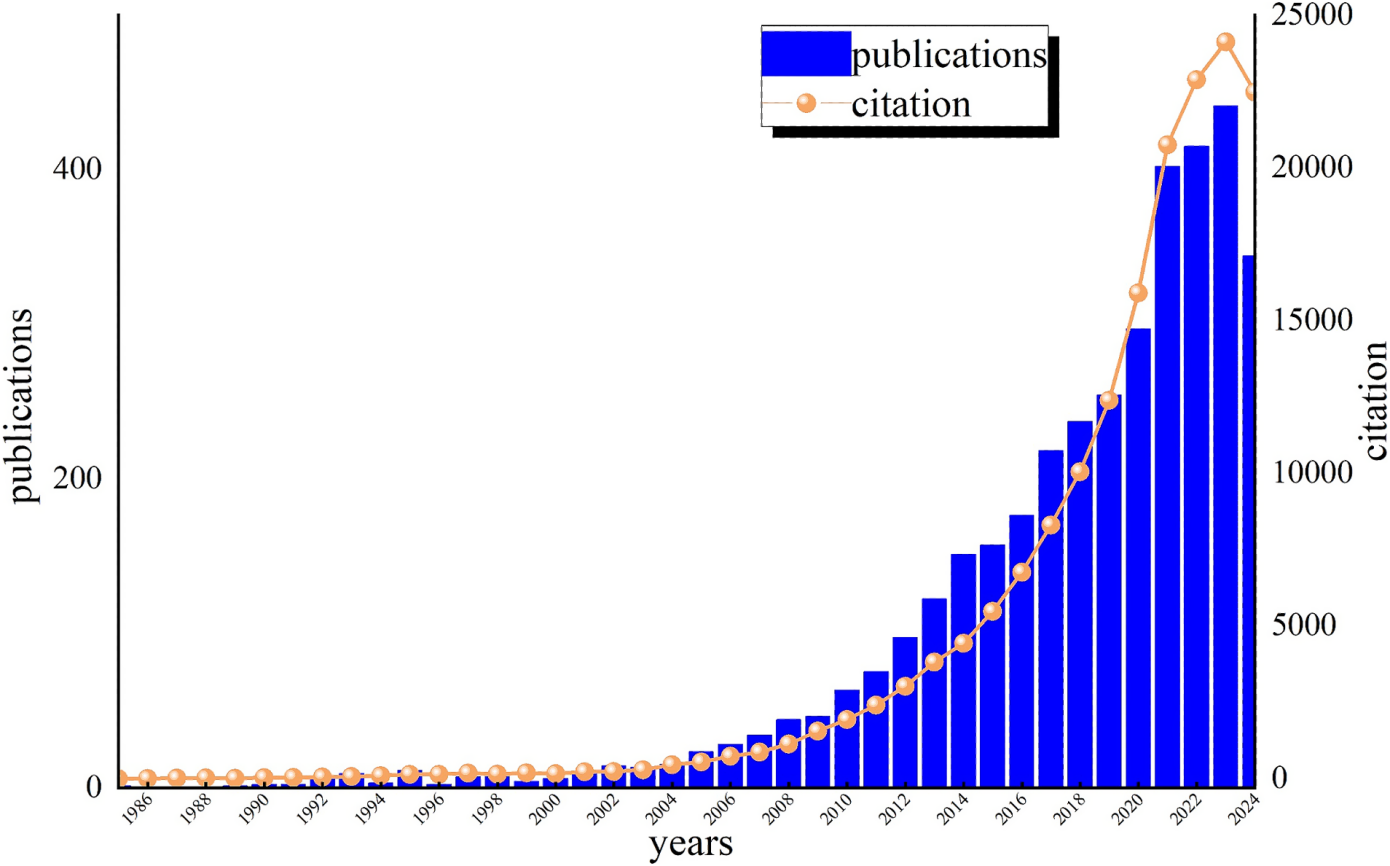
Annual publications and citations of cognitive impairment and immune research on the Web of Science (WOS).

Plotting the distribution of literature over time can effectively evaluate the state of research in this discipline and further predict its dynamics and trends. Figure 2 displays the annual distribution of literature related to cognitive impairment and immune research on the WoS for the past 40 years. From the trends in publication and citation, it is evident that the number of citations for these articles has increased steadily year by year. We divided the research into three phases, reflecting annual publication trends. From 1985 to 2000, the field saw limited development, with only ten articles published per year, marking the “preparation period.” The second phase (2001 to 2020) exhibited a gradual upward trend with a moderate growth curve. The third phase (2021 to 2024) experienced a surge, surpassing 400 annual publications and reaching 1,602 articles (Figure 2), indicating that cognitive impairment and immune research has entered a rapid development stage. The increase in publications from 2021 to 2024 may be attributed to growing attention to neuroimmune mechanisms during the COVID-19 pandemic. This trend is likely to continue in the coming years as researchers continue to uncover the complex relationships between cognitive health and immune function, which may lead to new breakthroughs and treatments for cognitive impairment.

### 3.2 Analysis of article output characteristics

#### 3.2.1 Co-countries analysis

**Figure 3:**
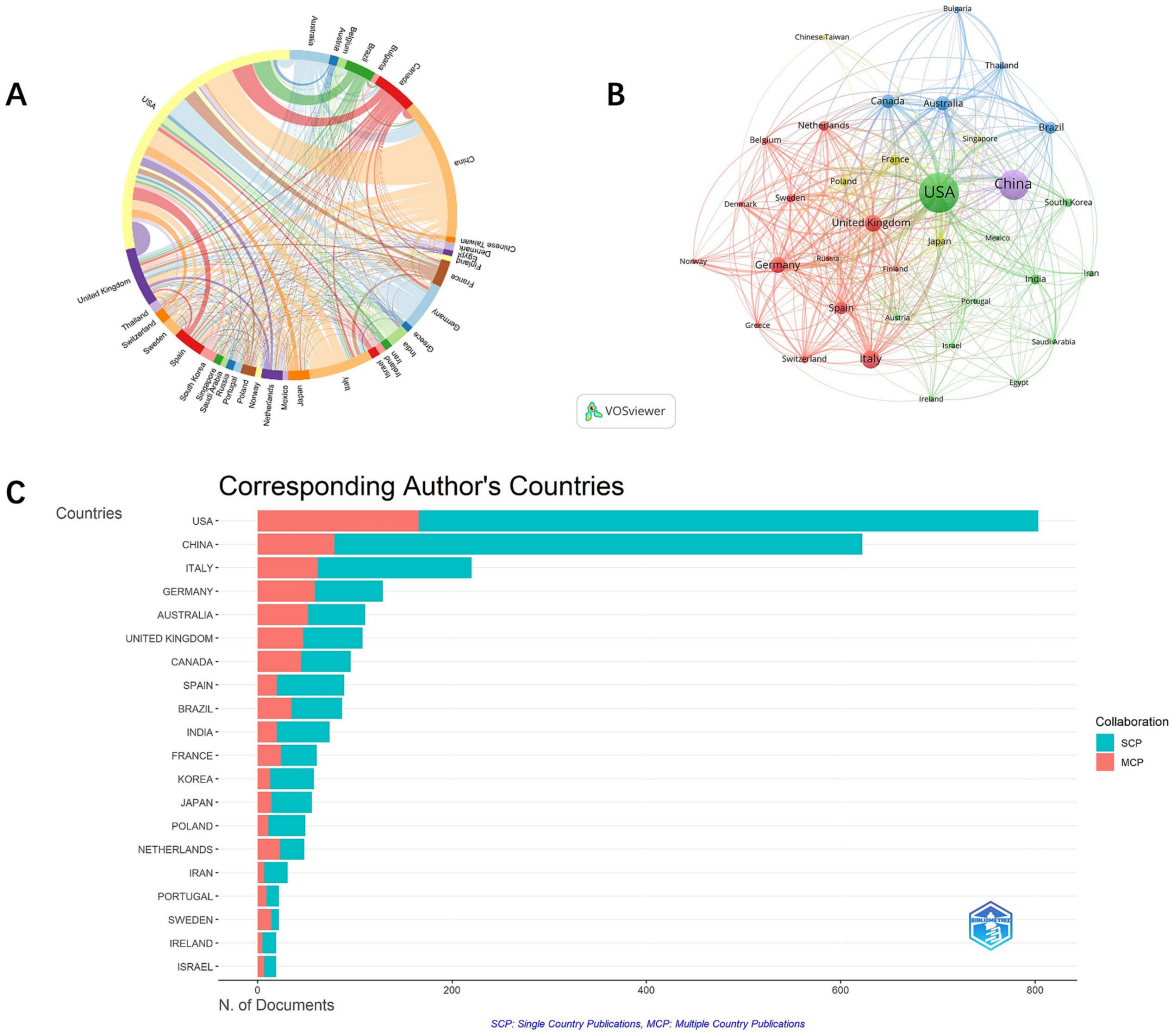
Chord diagram of countries co-authorship analysis. **(A)**. Network visualization map of countries co-authorship analysis. **(B)**. The number of citations and publications in the top 20 cited countries **(C)**.

Analysis of co-authorship involving countries, institutions, and authors facilitates the identification of potential partners, providing valuable insights for acquiring academic resources, fostering scholarly collaborations, and evaluating academic outcomes. A total of 58 countries actively contribute to cognitive impairment and immune research. Key contributors include the USA (1,419) and China (726) (Figure 3A). Publications from other countries or regions numbered fewer than 350, including Italy (314), the United Kingdom (290), Germany (236), and Canada (208). The USA dominates with the highest citations (88,373) and total link strength (TLS, 821). International collaboration shows relatively fewer joint publications (26.11%) (Figure 3B), with USA-centric collaborations occupying nine of the top 10 partnership positions, mainly with China.

#### 3.2.2 Co-institutions analysis

**Figure 4:**
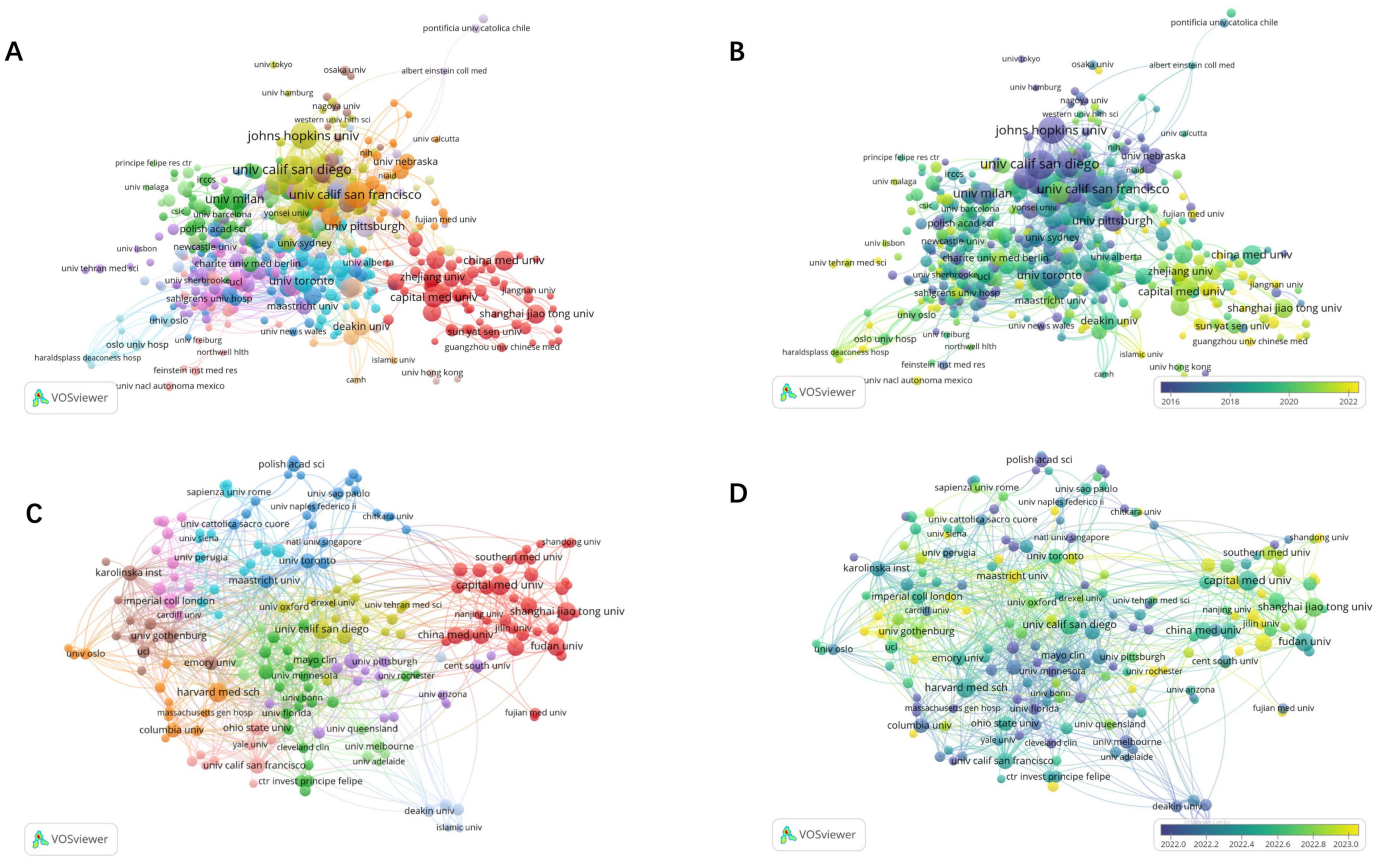
Network visualization map of institutions co-authorship analysis from 1985 to 2024 **(A)**. Overlay visualization map of institutions co-authorship analysis from 1985 to 2024 **(B)**. Network visualization map of institutions co-authorship analysis from 2021 to 2024 **(C)**. Overlay visualization map of institutions co-authorship analysis from 2021 to 2024 **(D)**.

A total of 4,418 institutions were included in the analysis, with 465 institutions having a minimum publication output of 5. The top ten contributors are predominantly from the USA. The University of California, San Diego leads with 80 articles and the highest citations (6,027). Despite ranking third in output, the University of California, San Francisco excels in TLS (200) (Table 1; Figures 4A,B. Five Chinese institutions are among the top 20 institutions in terms of contributions. However, most of them have relatively lower citation rates, with the exception of Shanghai Jiao Tong University (35 articles, 1,914 citations) and the Chinese Academy of Sciences (32 articles, 1,652 citations). Despite high publication output, Chinese institutions exhibited lower citation rates, possibly due to more recent publication dates, language barriers, and limited international collaborations. As shown in Figures 4C,D, from 2021 to 2024, a total of 2,715 institutions published articles, with 195 institutions having a minimum publication count of more than 5 articles. The analysis shows that institutions in the United States still have the highest publication numbers, followed by those in China and Europe (Table 2). Among them, the publications of Chinese institutions are relatively recent.

**Table 1:** Characteristics of the top 20 institutions based on publications from 1985 to 2024.

Rank	Institution	Documents	Citations	Total link strength	Country
1	Univ Calif San Diego	80	6,027	171	USA
2	Johns Hopkins Univ	75	5,627	173	USA
3	Univ Calif san Francisco	70	5,334	200	USA
4	Univ Calif Los Angeles	56	4,396	117	USA
5	Univ Milan	50	1993	59	Italy
6	Harvard Med Sch	48	2,842	159	USA
7	Univ Toronto	45	2,575	114	Canada
8	Univ Illinois	44	3,558	82	USA
9	Univ Pittsburgh	44	4,070	80	USA
10	Capital Med Univ	42	679	43	China
11	Karolinska Inst	42	1,682	128	USA
12	Stanford Univ	40	3,183	91	USA
13	Ohio State Univ	38	1873	47	USA
14	China Med Univ	37	520	11	China
15	Shanghai Jiao Tong Univ	35	1914	41	China
16	Fudan Univ	34	663	33	China
17	Chinese Acad Sci	32	1,652	63	China
18	Columbia Univ	32	1,372	65	USA
19	Harvard Univ	32	2,732	100	USA
20	Washington Univ	32	3,232	87	USA

**Table 2:** Characteristics of the top 20 institutions based on publications from 2021 to 2024.

Rank	Institution	Documents	Citations	Total link strength	Country
1	Capital Med Univ	28	428	25	China
2	Harvard Med Sch	25	501	48	USA
3	Shanghai Jiao Tong Univ	25	397	22	China
4	Fudan Univ	23	282	21	China
5	Univ Calif San Diego	23	261	25	USA
6	Zhejiang Univ	21	304	17	China
7	China Med Univ	20	219	3	China
8	Chinese Acad Sci	19	227	33	China
9	Univ Calif san Francisco	19	645	41	USA
10	Mayo Clin	18	414	30	USA
11	Nanjing Med Univ	18	174	21	China
12	Karolinska Inst	17	242	56	Sweden
13	Stanford Univ	17	1,019	24	USA
14	Imperial Coll London	16	271	35	United Kingdom
15	Columbia Univ	15	208	23	USA
16	Johns Hopkins Univ	15	123	37	USA
17	Ohio State Univ	15	245	11	USA
18	Univ Toronto	15	182	34	Canada
19	Washington Univ	15	330	25	USA
20	Emory Univ	14	211	28	USA

#### 3.2.3 Co-author analysis

**Figure 5:**
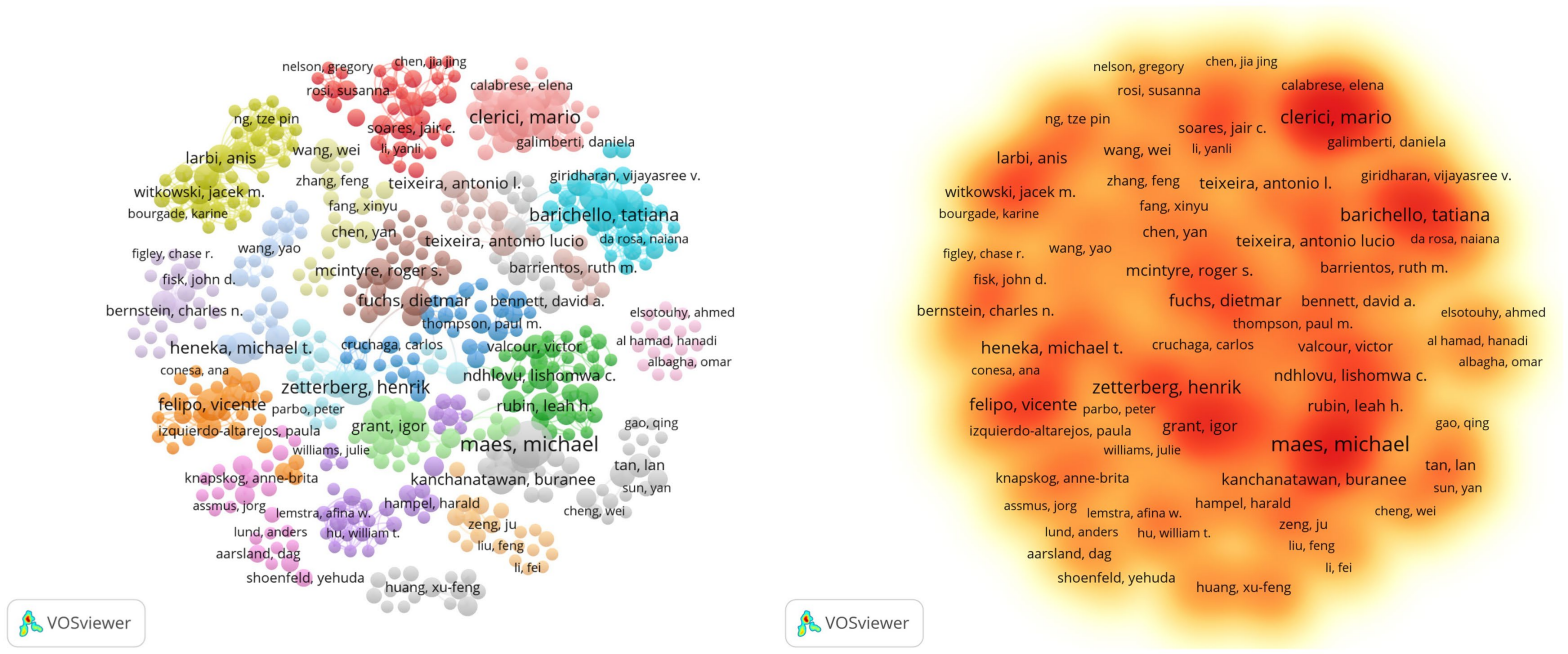
Network visualization map and density visualization map of authors citation analysis.

**Figure 6:**
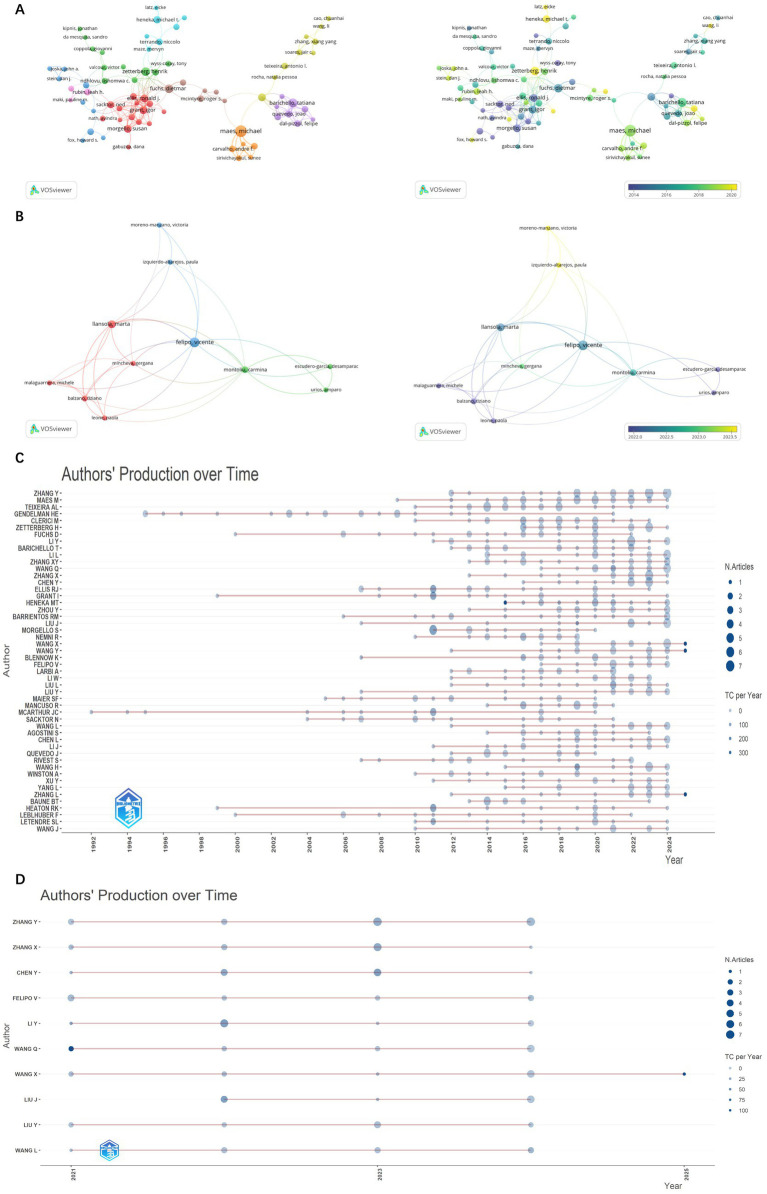
Network visualization map and overlay visualization map of authors citation analysis from 1985 to 2024 **(A)**. Network visualization map and overlay visualization map of authors citation analysis from 2021 to 2024 **(B)**. The number of citations and publications in the top 50 cited authors from 1992 to 2024 **(C)**. The number of citations and publications in the top 10 cited authors from 2021 to 2024 **(D)**.

This analysis, as shown in Supplementary Table S1 and Figure 5, reveals 21,430 contributors, with 193 authors having over five articles. Notable authors include Michael Maes (the highest articles: 29; citations: 1,465; TLS: 38), Mario Clerici (articles: 21; citations: 989; TLS: 109), and Michael T. Heneka (articles: 12; citations: 1,552; TLS: 5). Additionally, Tony Wyss-Coray, Michael T. Heneka, and Michal Schwartz, despite a publication count of only 6–12 articles, rank among the top three in citation frequency, showcasing their high-quality contributions. Michael Maes focuses his research on inflammation-related psychiatric disorders such as major depressive disorder, bipolar disorder, and schizophrenia, as well as cognitive impairments associated with these conditions (Al-Hakeim et al., 2018; Maes and Kanchanatawan, 2021). Mario Clerici specializes in investigating the impact of inflammatory mediators, such as cytokines and chemokines, on cognitive function in both normal and neurodegenerative disease states, with his research spanning the fields of molecular biology and genetics (Krasemann et al., 2017; Mancuso et al., 2019, 2020). Heneka emphasizes that neuroinflammation induced by the immune system may be a significant contributor to the onset of neurodegenerative cognitive impairments, in which microglia play a dual role (Heneka et al., 2015). He particularly focuses on microglia and the NLRP3 inflammasome as potential therapeutic targets, exploring their protective effects in this context. Based on Figure 6C, it is evident that while some high-producing authors have experienced a decline in their output in recent years, Heneka has consistently maintained a high level of quality output since 2014. This demonstrates his continued dedication and credibility in his field.

During 2021–2024, there are 10,988 contributors, with 174 authors having over three articles. Based on the analysis of publication number, there are some differences compared to the data from 1985 to 2024 (as shown in Figures 6A,B and Supplementary Table S2). Vicente Felipo occupies a central position in author collaboration, based on Figure 6D, he has maintained a stable publication output since 2021, through whom many authors are able to co-publish papers. Meanwhile, we can observe that although the number of publications is not high, Victoria Moreno-Manzano, Paula Izquierdo-Altarejos, and Mincheva, Gergana, who are from the same institution and collaborate with Vicente Felipo have published very recent work. They all mainly studied the reversal effect of extracellular vesicles on neuroinflammation in metabolic and neurodegenerative diseases, as well as their improvement on cognition (Wang et al., 2024). They also proposed that neuroinflammation not only leads to neuronal damage and death but also affects the connectivity and function of neural networks.

Co-citation analysis, a methodology in which a third author or paper concurrently cites two authors or papers, elucidates these intricately interwoven associations. A total of 194 authors who published more than 5 papers have been co-cited, illustrating the clustering information of co-cited authors analyzed using CiteSpace. By analyzing co-cited author clusters, researchers can discern highly co-cited scholars and their impact within a specific field. We used CiteSpace to analyze the author co-citation clustering for the two time periods of 1985–2024 and 2021–2024, as shown in Supplementary Tables S3, S4. Across these timeframes, it is evident that HIV-associated neurocognitive disorders garnered brief attention. Over a longer timespan (such as the entire 1985–2024 period), AD, microglia, T cells, and the NLRP3 inflammasome have consistently been research hotspots. Research in these areas may have continued to deepen, forming stable research communities and knowledge systems.

Emerging research areas: In the more recent timeframe (2021–2024), areas such as gut microbiota and trem2 have begun to receive increasing attention. These new areas may have introduced new research directions and academic controversies, potentially driving the development of related fields.

### 3.3 Analysis of the journals

The criteria for assessing a journal’s influence include publication volume, citation frequency, and Impact Factor (IF). Among the top ten journals, only three have an IF over 10: NATURE (IF 50.5), SCIENCE (IF 44.8), and NEURON (IF 14.7). J neurosci leads in citations (6531).

The purpose of co-citation analysis is to identify the papers frequently cited and the journals publishing these papers within a research field. By utilizing VOSviewer to create a co-citation map of journals, we set a threshold of a minimum of 500 co-citations for each journal, resulting in the selection of 119 journals for the co-citation analysis of cited journals. The final co-citation relationship diagram is presented in Table 3.

**Table 3:** The top 10 journals contributed to publications on immune and cognitive impairment research.

Rank	Journal	Documents	Citation	Total link strength	JCR/impact factor (2023)	Country
1	PLoS One	2,164	5,118	354,715	2.9	USA
2	P Natl Acad Sci USA	2091	5,408	434,781	9.4	USA
3	J Neurosci	1897	6,531	545,077	4.4	USA
4	Nature	1788	4,758	406,610	50.5	ENGLAND
5	Neurology	1718	5,723	381,560	8.4	USA
6	Brain Behav Immun	1,682	4,793	323,206	8.8	USA
7	Science	1,600	3,552	297,048	44.8	USA
8	J Neuroinflamm	1,484	3,627	295,725	9.3	ENGLAND
9	Neurobiol Aging	1,294	3,697	312,281	3.7	ENGLAND
10	Neuron	1,251	2,876	266,911	14.7	USA

**Figure 7:**
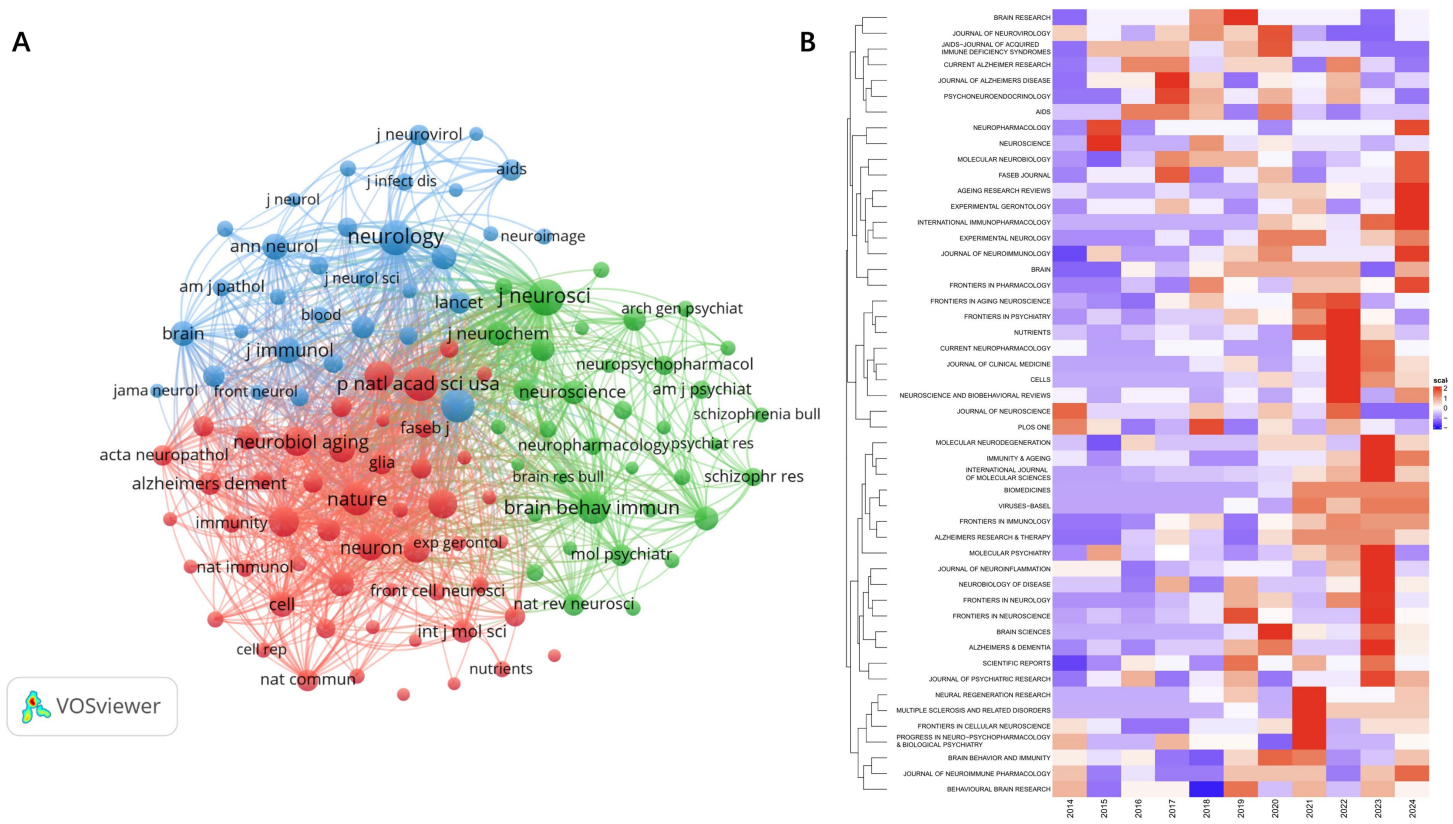
Network visualization map of source citation analysis **(A)**. Heatmap of source citation analysis **(B)**.

The co-citation network mainly consists of three clusters, corresponding to the three colors in Figure 7A. The top three journals in terms of citation count are J Neurosci (6,531 citations), Neurology (5,723 citations), and P Natl Acad Sci USA (5,408 citations). Among the three clusters, the journals in the red cluster primarily focus on aging, dementia, and biochemistry, while the journals in the green cluster mainly concentrate on the pathophysiology and pharmacology related to psycho-behavioral impairments. The journals heatmap indicates that the co-cited journals from 2021 to 2024 are more concentrated than before and appear in cluster form. In 2021, published articles emphasized the diversification of research methods and the application of new technologies. The magazines in 2022 covered more cross-disciplinary research across multiple professional fields, highlighting the integration of basic research and clinical applications. In 2023, while continuing the trend of multidisciplinary intersection, the published articles focused on the relationship between immunity and cognition in disease states, with an emphasis on exploring mechanisms. In 2024, the magazines were more focused on aging-related topics and continued to explore the relationship between neuro-immunity in disease models (Figures 7A,B).

### 3.4 Analysis of keywords

**Figure 8:**
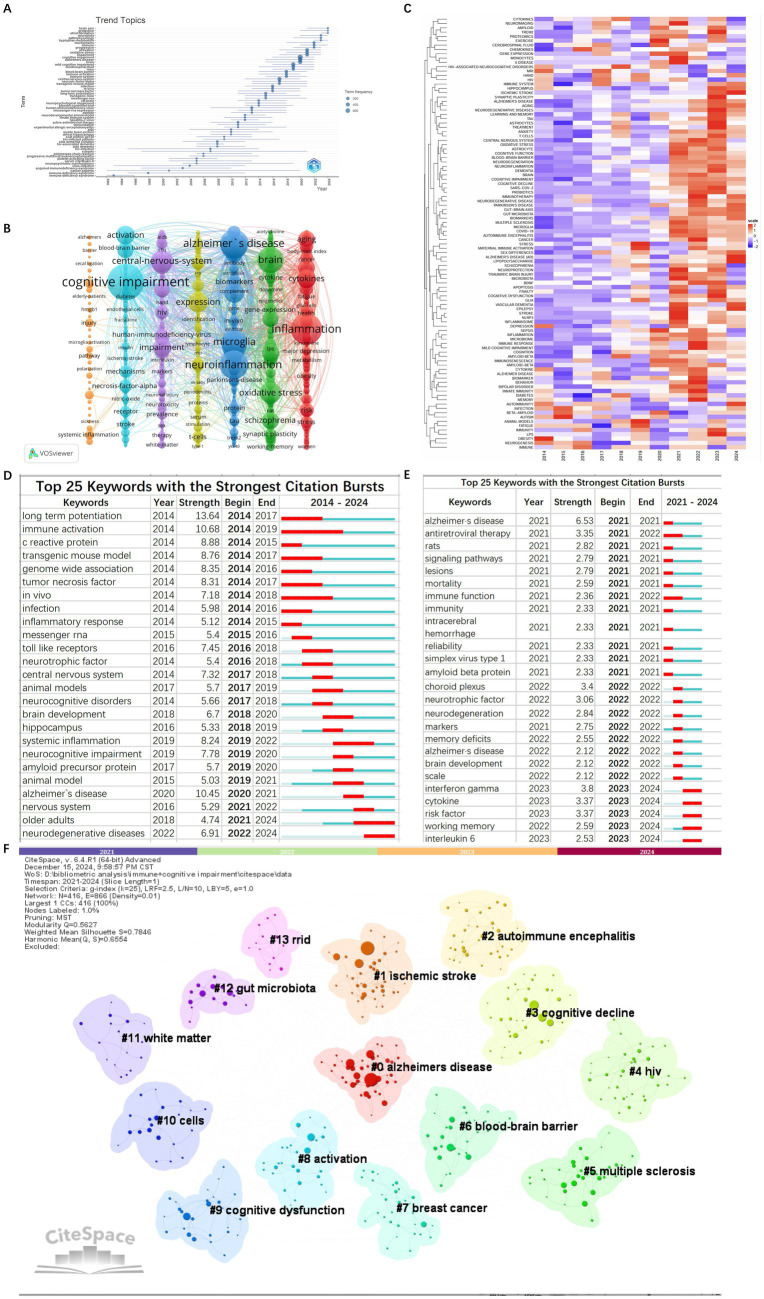
Bibliometrix keyword trend analysis in the field of immune and cognitive impairment studies **(A)**. Keywords co-occurrence network visualization map in the field of immune and cognitive impairment studies **(B)**. Heatmap of keywords analysis **(C)**. The top 25 keywords with the strongest citation bursts from 2014 to 2024 **(D)**. The top 25 keywords with the strongest citation bursts from 2021 to 2024 **(E)**. The cluster visualization map of keywords with the strongest citation bursts **(F)**.

Keywords play a pivotal role in summarizing the themes and content of articles. Based on bibliometric keyword trend analysis it can be seen that the keywords have become prominent and concentrated during the period from 2014 to 2024 (Figure 8A).

The extraction of 13,988 keywords unveiled the top three: cognitive impairment (971), inflammation (716), and neuroinflammation (604) (Figure 8B).

We have analyzed the keywords over the past decade. The keyword heatmap reveals that, in addition to the persistent focus on cognitive impairment, neuroinflammation, and AD, new keywords such as ischemic stroke, astrocytes, neuroprotection, immunotherapy, and vascular dementia have emerged from 2021 to 2024 (Figure 8C).

Keyword burst detection identifies terms with markedly increased frequencies signaling emerging research directions. Burst analysis identifies 25 keywords withrobust citation bursts including long-term potentiation (13.642014–2017) immune activation (10.682014–2019) and alzheimer’s diseases (10.452020–2021). In the recent 4 years (2021–2024) keywords with significant bursts include alzheimers diseases (6.532021) choroid plexus (3.42022) antiretroviral (3.352021–2022) cytokine (3.372023–2024) risk factor (3.372023–2024) and working memory (2.592023–2024) (Figures 8D–F) indicating that future research may increase the focus on vascular factors. The transition from early mechanistic terms such as ‘long-term potentiation’ to immune-related keywords like ‘cytokines’ further reflects a thematic shift in the field—from classical neurophysiological mechanisms to immune-mediated models of cognitive impairment.

**Figure 9:**
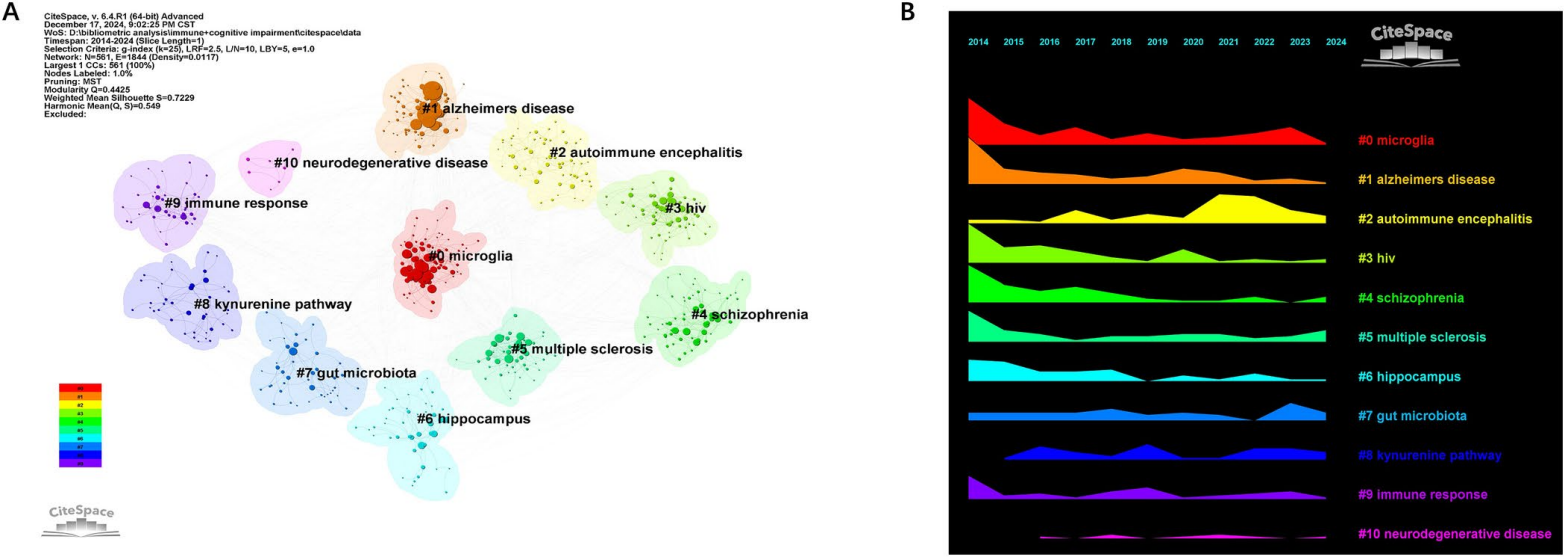
The cluster visualization map of keywords analysis **(A)**. The timeline chart of keywords analysis **(B)**.

Cluster analysis of keywords enables a comprehensive and precise examination of relationships among keywords. Cluster analysis generated reveals 10 keyword clusters using CiteSpace: #0 microglia, #1 Alzheimer’s disease, #2 autoimmune encephalitis, #3 hiv, #4 schizophrenia, #5 multiple sclerosis, #6 hippocampus, #7 gut microbiota, #8 kynurenine pathway, #9 immunne response, #10 neurodegenerative disease. #0, #1, and #2 emerged as the top 3 clusters. Figures 9A,B illustrate the developmental trajectory of keyword clusters, highlighting ongoing advancements in clusters #5, #7, and #8. These continuous advancements underscore the current dynamism of these research domains in immune and cognitive impairment studies.

### 3.5 Seminal literature: landmark research

Highly cited literature often signifies impactful and innovative research in its field. Analyzing these studies provides insights into advancements, trends, and future directions in immune and cognitive impairment studies.

Scrutinizing the countries, institutions, and authors associated with this literature identifies key contributors in the immune and cognitive impairment domain, aiding global research understanding and fostering future collaborations.

Among the top 15 cited works, there are 6 reviews, 6 articles, 1 meta-analysis, and 2 comments. The USA is the primary contributor (8), followed by Germany (3) and Israel (2). Table 4 highlights the top 15 articles, including Heneka MT’s 2015 review in *LANCET NEUROL,* which garnered the highest number of citations (193). It reviewed that the pathogenesis of Alzheimer’s disease is not restricted to the neuronal compartment but includes strong interactions with immunological mechanisms in the brain. Modulation of risk factors and targeting these immune mechanisms could lead to future therapeutic or preventive strategies for Alzheimer’s disease. Other highly cited articles predominantly discuss the role of neuroinflammation in various neurocognitive disorders, including Alzheimer’s disease, addressing immune activation, neuroinflammatory mechanisms, TREM2, NLRP3, cognitive impairment, and their relationship with disease progression.

**Table 4:** Characteristics of the top 15 articles based on citations from 1985 to 2024.

Rank	Title	Year	Citation	Total link strength	Author	Institute	Country	Journal	Type
1	Neuroinflammation in Alzheimer's disease	2015	193	705	Heneka MT	University Hospital Bonn	Germany	Lancet Neurol	Review
2	From inflammation to sickness and depression: when the immune system subjugates the brain	2008	139	178	Robert Dantzer	University of Illinois	USA	Nat Rev Neurosci	Review
3	Inflammation and Alzheimer's disease	2000	132	449	Akiyama H	Sun Health Research Institute	USA	Neurobiol Aging	Review
4	Updated research nosology for HIV-associated neurocognitive disorders	2007	129	165	Antinori A	Instituto di Ricovero e Cura a Carattere Scientifico (IRCCS)	Italy	Neurology	Review
5	HIV-associated neurocognitive disorders persist in the era of potent antiretroviral therapy: CHARTER Study	2010	122	167	Heaton RK	University of California	USA	Neurology	Clinical research
6	NLRP3 is activated in Alzheimer’s disease and contributes to pathology in APP/PS1 mice	2013	110	527	Heneka MT	University of Bonn	Germany	Nature	Research
7	Complement and microglia mediate early synapse loss in Alzheimer mouse models	2016	107	451	Hong S	Boston Children's Hospital and Harvard Medical School	USA	Science	Research
8	A Unique Microglia Type Associated with Restricting Development of Alzheimer's Disease	2017	103	470	Keren-Shaul H	Weizmann Institute of Science	Israel	Cell	Research
9	Neurotoxic reactive astrocytes are induced by activated microglia	2017	103	337	Liddelow SA	Stanford University	USA	Nature	Research
10	"Mini-mental state". A practical method for grading the cognitive state of patients for the clinician	1975	101	168	Folstein MF	The New York Hospital-Cornell Medical Center	USA	J Psychiat Res	Research
11	TREM2 variants in Alzheimer's disease	2013	101	565	Guerreiro R	University College London (UCL) Institute of Neurology	UK	New Engl J Med	Meta
12	The amyloid hypothesis of Alzheimer's disease: progress and problems on the road to therapeutics	2022	99	379	Hardy J	National Institute on Aging	USA	Science	Review
13	The diagnosis of dementia due to Alzheimer's disease: recommendations from the National Institute on Aging-Alzheimer's Association workgroups on diagnostic guidelines for Alzheimer's disease	2011	96	225	Mckhann GM	Johns Hopkins University School of Medicine	USA	Alzheimers Dement	Comments
14	Immune attack: the role of inflammation in Alzheimer disease	2015	92	397	Heppner FL	Charité - Universitätsmedizin Berlin	Germany	Nat Rev Neurosci	Review
15	Immune modulation of learning, memory, neural plasticity and neurogenesis	2011	92	166	Yirmiya R	The Hebrew University of Jerusalem	Israel	Brain Behav Immun	Review

They also explore the impact of various factors, such as age, activation of the peripheral immune system, and immune cell function, on neuroinflammation and cognitive function.

### 3.6 Analysis of co-cited references

#### 3.6.1 Clusters and timeline of research

**Figure 10:**
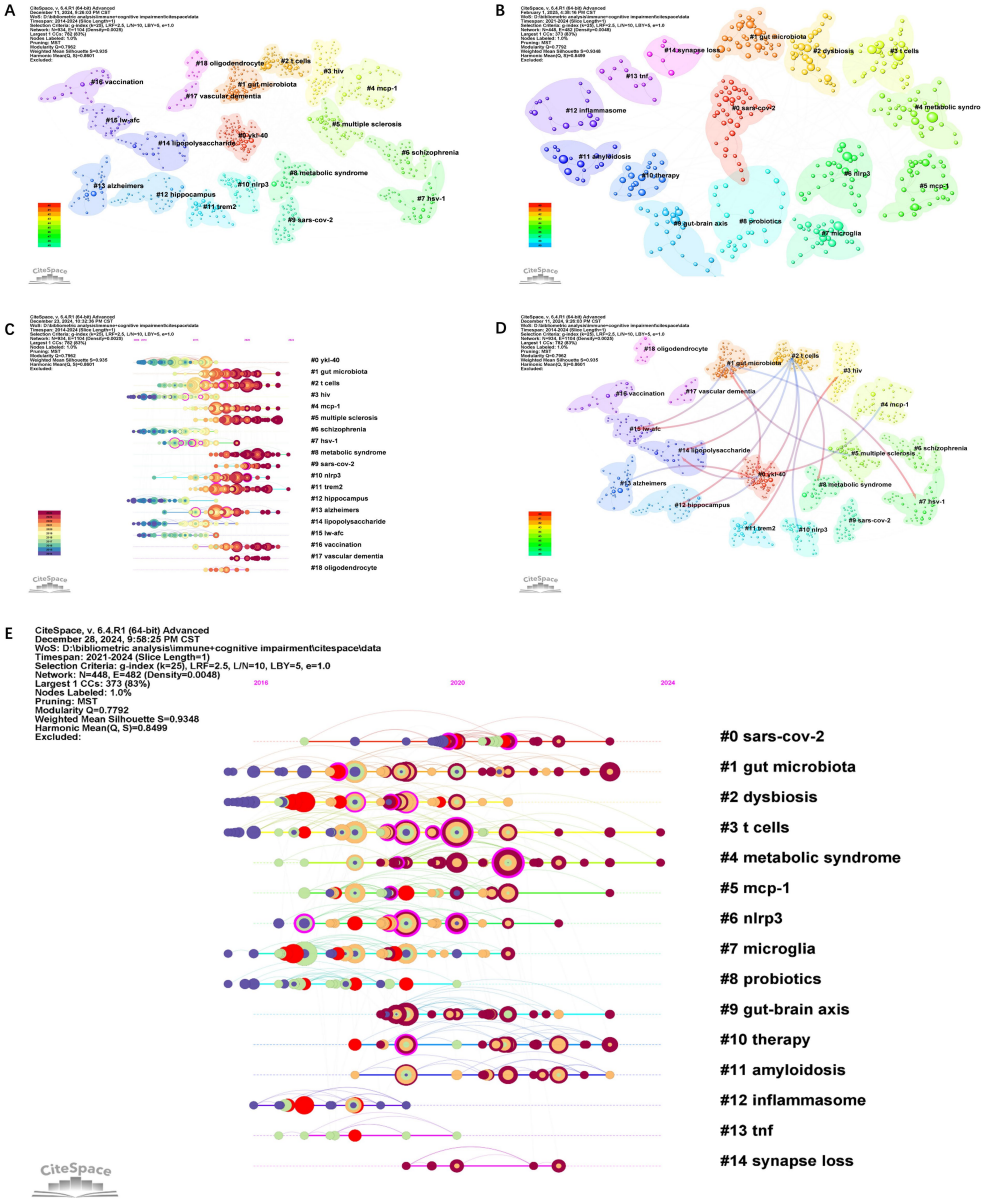
The co-citation cluster visualization map of references from 2014 to 2024 **(A)**. The co-citation cluster visualization map of references from 2021 to 2024 **(B)**. The co-citation network visualizations map of keywords from 2014 to 2024 **(C)**. The co-citation cluster visualization map of references from 2014 to 2024 **(D)**. The co-citation network visualizations map of keywords from 2021 to 2024 **(E)**.

Co-citation reflects the referencing of two or more papers by other scholarly works, showcasing their interconnectedness within the research domain (27). Co-citation analysis elucidates foundational knowledge and the frontiers of academic research, enhancing our understanding of the field’s structure and evolution. CiteSpace was employed for a 2014–2024 co-citation analysis, resulting in 18 principal clusters. The clustering results (*Q* = 0.79, *S* = 0.93) highlight reliability (Figure 10A). The largest cluster is #0 ykl40. There are 13 principal clusters for the 2021–2024 co-citation analysis (*Q* = 0.77, *S* = 0.93) (Figure 10B). When comparing the two sets of data, the gut microbiota, T cells, metabolic syndrome, MCP-1, and NLPR3 have all garnered significant attention. The emergence of terms such as “microglia” and “inflammasome” during this period indicates an increased interest in studying the role of microglia in immunity and cognitive impairments from 2021 to 2024. As immune cells in the brain, microglia’s role in neuroinflammation, neurodegenerative diseases, and other pathological processes related to cognitive impairments may become a research hotspot. Figure 10C shows the temporal evolution of the main clusters. It is evident that clusters #8 and #11 have received sustained attention. Outlining their critical research focuses, most of the literature in Cluster 8 on metabolic syndrome consists of reviews. These discuss the involvement of neuroinflammation and microglial activation in the pathogenesis of Alzheimer’s disease and other neurodegenerative disorders. They emphasize the role of microglia, the resident immune cells of the brain, in responding to neuronal damage and contributing to inflammation. Several articles mention the role of astrocytes, another type of glial cell, in CNS inflammation and their crosstalk with microglia and other cells. The interaction between astrocytes and microglia is highlighted as a crucial aspect of neuroinflammatory processes. In addition, the role of inflammation and gut microbiota in mediating inflammatory pathways in AD is also explored.

The cluster#11 trem2 is a recent cluster, with the average publication year of the literature being 2018. Nine out of the top ten articles in the #11 cluster have exhibited citation bursts; three of these articles have continued to receive attention until 2024, indicating that this cluster will become an emerging trend in the field. These articles are predominantly published in high-impact journals such as Nature and Cell. Most of the literature in Cluster 11 primarily focused on the genetic and molecular aspects of Alzheimer’s disease (AD), with a strong emphasis on microglia, immunity, and genetic risk loci. Mathys H presents a single-cell transcriptomic analysis of Alzheimer’s disease, providing insights into the gene expression profiles of different cell types, including microglia, in AD brains (Mathys et al., 2019). Jonathan Kipnis and others highlight the role of the TREM2-APOE pathway in driving microglial dysfunction in AD and other neurodegenerative diseases (Gratuze et al., 2023). David H. Holtzman and others present the complexity and dynamic nature of microglial function in health and disease (Chen et al., 2023; Parhizkar et al., 2023). Some articles revisit or reevaluate the amyloid hypothesis (the idea that the accumulation of amyloid-beta plaques in the brain is a central cause of AD) (Pimplikar, 2009; Armstrong, 2011), while others explore alternative or additional pathways, such as tau pathology, lipid processing, and immune dysfunction (Cao et al., 2018; Yamazaki et al., 2019). They implicate microglial-mediated innate immunity in the disease process and highlight the importance of microglial function and dysfunction in neurodegeneration. Genetic meta-analyses analyze large datasets to uncover genetic variations that increase the risk of developing AD and have identified new risk loci for Alzheimer’s disease (Lambert et al., 2013a,b; Sims et al., 2017; Lancaster et al., 2019).

#### 3.6.2 Most co-cited papers

Table 5 highlights the top 10 articles, including those of cluster #2 T cells dominate, with other clusters such as #4 MCP1, #8 metabolic syndrome, #10 NLRP3, #11 Trem 2, and #13 Alzheimer’s. Cluster dependencies show that Cluster #2 T cells is a relatively new group that cites the most literature from other clusters, such as clusters #0, #8, #11, #12, #14, and #15. The most highly cited literature, Heneka MT’s 2015 review, also originates from this cluster. Figure 10D shows that cluster #1 gut microbiota cites articles from clusters #0, #7, and #15. The cluster with the most citations is cluster #0 YKL-40, whose articles are cited by clusters #1, #2, #4, and #13. #0 YKL-40 and #15 lw-afc are cited by clusters #1 gut microbiota and #2 T cells. Heneka MT’s 2015 review in LANCET NEUROL and Keren-Shaul H’s 2017 article in CELL garnered the highest number of citations (8,468) (Table 5). Heneka MT reviewed that Alzheimer’s disease pathogenesis is not restricted to the neuronal compartment but includes strong interactions with immunological mechanisms in the brain. Modulating risk factors and targeting these immune mechanisms could lead to future therapeutic or preventive strategies for Alzheimer’s disease. Keren-Shaul et al. (2017) affirmed that immunohistochemical staining of mouse and human brain slices reveals a microglial type associated with neurodegenerative diseases (disease-associated microglia, DAM) containing Aβ particles. Single-cell analysis shows that DAM activation occurs in a Trem2-independent and then Trem2-dependent two-step process, potentially restricting neurodegeneration (Zhou et al., 2020). Other highly cited articles broadly cover several research aspects and questions related to dementia and neuroinflammation. Inflammation is a recurring theme throughout, highlighting its pivotal role in AD and other neurodegenerative diseases. Specifically, they delve into five points:

**Table 5:** Characteristics of the top 10 most co-cited articles.

Rank	Title	Year	Citation	Author	Institute	Country	Journal	Type	Total link strength
1	Neuroinflammation in Alzheimer’s disease	2015	84	Heneka MT	University Hospital Bonn	Germany	Lancet Neurol	Review	2
2	A Unique Microglia Type Associated with Restricting Development of Alzheimer’s Disease	2017	68	Keren-Shaul H	Weizmann Institute of Science	Israel	Cell	Research	13
3	IFN-γ Production by Amyloid β–Specific Th1 Cells Promotes Microglial Activation and Increases Plaque Burden in a Mouse Model of Alzheimer’s Disease	2013	61	Browne TC	Trinity College Dublin	Ireland	The Journal of Immunology	Research	2
4	Immune attack: the role of inflammation in Alzheimer disease	2015	58	Heppner FL	University of Zürich	Switzerland	Nat Rev Neurosci	Review	2
5	Neurotoxic reactive astrocytes are induced by activated microglia	2017	55	Liddelow SA	Johns Hopkins University School of Medicine	USA	Nature	Research	4
6	Neuroinflammation and microglial activation in Alzheimer disease: where do we go from here?	2021	54	Leng FD	Hammersmith Hospital Campus	United Kingdom	Nat Rev Neurol	Review	8
7	Complement and microglia mediate early synapse loss in Alzheimer mouse models	2016	49	Hong S	Broad Institute of MIT and Harvard	USA	Science	Research	2
8	NLRP3 inflammasome activation drives tau pathology	2019	47	Ising C	University of Massachusetts Medical School, Worcester	USA	Nature	Research	10
9	NIA-AA Research Framework: Toward a biological definition of Alzheimer’s disease	2018	43	Jack CR	Brigham and Women’s Hospital	USA	Alzheimers Dement	Review	2
10	Genetic meta-analysis of diagnosed Alzheimer’s disease identifies new risk loci and implicates Aβ, tau, immunity and lipid processing	2019	42	Kunkle BW	University of Miami Miller School of Medicine	USA	Nat Genet	Meta	11

Immune Cells in AD: Studies examine the role of clonally expanded CD8 T cells and microglia in patrolling the cerebrospinal fluid and neuroinflammatory processes. The involvement of TREM2 variants and neutrophils in AD is also explored, highlighting the complexity of the immune system in the disease.

Neuroinflammation and Microglial Activation: Multiple articles focus on microglial activation and neuroinflammation, including their roles in synapse loss, tau pathology, and the transcriptional phenotype of dysfunctional microglia. These studies aim to understand how neuroinflammatory processes contribute to dementia pathogenesis.

Biological Mechanisms and Genetic Risk Factors: Research frameworks and genetic meta-analyses are presented to define AD biologically and identify new risk loci. Topics such as Aβ, tau, immunity, and lipid processing are implicated in AD risk, providing insights into the disease’s underlying mechanisms.

Gut Microbiome and Brain Health: Alterations in the gut microbiome are associated with dementia, suggesting a gut–brain axis in the disease. Pro-inflammatory gut bacterial taxa and peripheral inflammation markers are linked to brain amyloidosis in cognitively impaired elderly, further supporting this connection.

Blood–Brain Barrier and Inflammation: The breakdown of the blood–brain barrier in AD and other neurodegenerative disorders is discussed, emphasizing inflammation as a central mechanism in AD pathogenesis. Many articles aim to bridge the gap between basic research and clinical applications, discussing potential therapeutic strategies and implications for future treatments.

#### 3.6.3 Burst analysis

**Figure 11:**
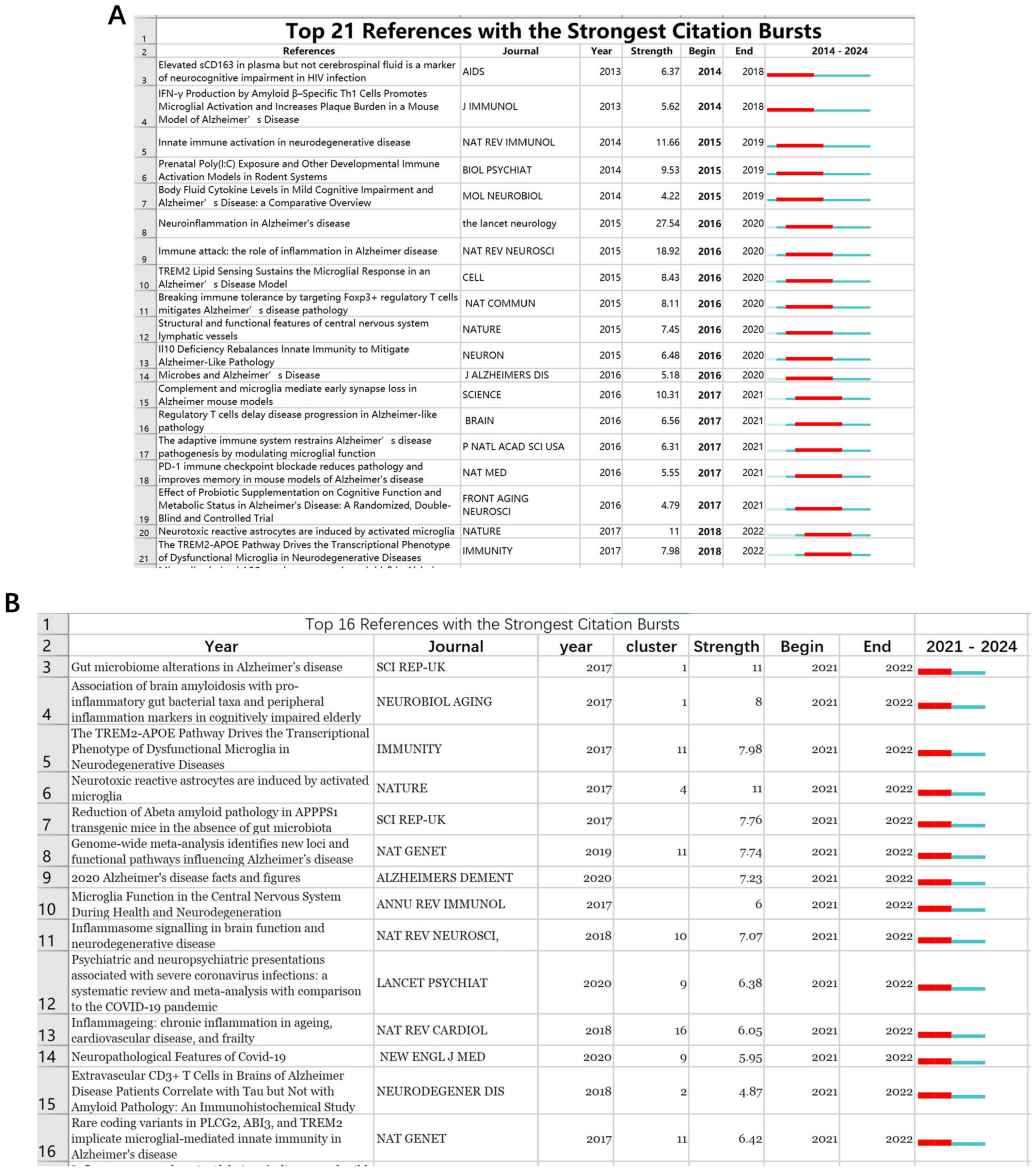
The top 25 references with the strongest citation bursts from 2014 to 2024 **(A)**. The top 16 references with the strongest citation bursts from 2021 to 2024 **(B)**.

The eruption of citations has provided a valuable means of observing the evolution of research focal points. A total of 567 references underwent citation bursts. Figure 11A displays the 21 articles with the highest citation burst strength during 2014–2024, underscoring the flux of comparable thematic trends. The article with the highest citation burst strength was Heneka MT’s 2015 review in *LANCET NEUROL*. In CiteSpace, from 2014 to 2024, 567 documents experienced citation bursts (Figure 11A displays only the top 21), with 8 in #2 T cells. From 2021 to 2024, 308 documents still experienced citation bursts (Figure 11B displays only the top 16), with 3 in #1 gut microbiota, indicating these areas as key current and future focuses of immune and cognitive impairment research.

## 4 Discussion

### 4.1 General information

This study conducted a systematic bibliometric analysis of the literature related to cognitive impairment and immunity from 1985 to 2024 by retrieving 3,737 documents (2,562 articles; 1,175 reviews) and 216,933 references.

Results of this study showed that researchers from 96 countries and regions engaged in immune system and cognitive impairment studies, highlighting the global prominence of this topic. The top ten countries in terms of publication output account for 70% of the total literature. The United States and China significantly outnumber other nations. The United States had the highest number of publications in the early stages, and its papers also had the highest citation counts. However, in recent years, Chinese authors have emerged with a significant number of publications, particularly in the field related to gut microbiota and cognition.

Universities played a pivotal role in this context. Among the top 10 institutions, a majority are situated in the USA. Among the top 20 publishing countries, five research institutions in China have distinguished themselves. An analysis of relevant publications from 2021 to 2024 shows that six out of the top 10 publishing institutions are from China. This indicates that China has shown tremendous potential in research on immunity and cognitive impairments in recent years.

In the long course of time, some authors may achieve remarkable accomplishments early in their careers but gradually lose momentum. However, Professor Heneka MT has consistently produced high-quality papers and maintained a focus on his research areas of neuroinflammation, microglia, and the NLRP3 inflammasome. This demonstrates his dedicated effort and expertise in this field and showcases his academic authority. Maes, Michael, and Zetterberg, Henrik, have consistently sustained the momentum of their research endeavors, boasting a considerable number of publications. Professor Michael Maes has dedicated himself to exploring psychiatry and molecular neuroscience, as well as long-COVID-associated mental ailments after 2021. Professor Henrik Zetterberg’s primary research interests encompass the role of neuroinflammation in the etiology of neurodegenerative diseases and the study of associated cerebrospinal fluid and blood biomarkers (Heneka et al., 2015; Zetterberg and Blennow, 2021). Post-2021, he has intensified his efforts on the early diagnosis of diseases and the clinical utilization of biomarkers, as well as the interplay between neurodegenerative diseases and other systemic conditions (Kelly et al., 2023; Hong et al., 2025; Pedersen et al., 2025). Professor Vicente Felipo has long concentrated his research on the association between metabolic diseases, neuroinflammation, and cognitive impairments (Mekhora et al., 2024; Sørensen et al., 2024). Nevertheless, in recent years, his attention has shifted to the function of cellular vesicles in this process, especially emphasizing the treatment of neuroinflammation and cognitive impairment-related diseases using extracellular vesicles (Upadhya and Shetty, 2019; Tang et al., 2020; Ruan et al., 2021; Yuan et al., 2021). The number of studies in the immune and cognitive impairment field has shown a significant growing trend, especially from 2021 to 2024, entering a rapid development stage, with 1,602 publications in 2024, and the number of citations of the literature has increased steadily year by year. This indicates that research on the relationship between cognitive impairment and immunity has attracted extensive attention in the academic community, and research enthusiasm continues to rise.

### 4.2 Critical research topics in the domain of immune and cognitive impairment, hotspots and frontiers

The analysis of keywords and references yields valuable insights into the primary research focus. In recent years, interferon gamma, cytokines, risk factors, working memory, interleukin-6, older adults, and neurodegenerative diseases have emerged as the strongest citation burst keywords. This suggests that immune regulators such as interferon gamma and interleukin-6, among other cytokines, may serve as risk factors for cognitive function in the elderly population and are closely related to the onset and progression of neurodegenerative diseases. Keyword clustering analysis indicated that each cluster had a clear theme and a certain depth. For example, the #7 gut microbiota cluster not only involves the association between changes in gut microbiota composition and cognitive impairment but also deeply explores the mechanisms by which gut microbiota affects neuroinflammation, neurotransmitter metabolism, and synaptic plasticity through the “gut–brain axis” (Gareau et al., 2011; Sampson et al., 2016; Vogt et al., 2017; Strandwitz, 2018; Cryan et al., 2019). Evidence suggests that early-life interactions between the microbiota and the immune system play a critical role in immune programming, which may have long-term consequences for brain development and cognitive function (Donald and Finlay, 2023). The #9 immune response cluster focuses on the immune response process in cognitive impairment, including research on “inflammatory factors,” “immune cell activation,” and “autoantibodies,” and deeply analyzes the dual role of the immune response in the occurrence and development of cognitive impairment, specifically how the imbalance of the normal immune response leads to neuropathological damage and cognitive decline (Heneka et al., 2015; Heppner et al., 2015; Hong et al., 2016; Schwartz and Deczkowska, 2016; Prinz and Priller, 2017; Dalmau and Graus, 2018). Additionally, there are close associations and synergies between different clusters. For example, the #0 microglia cluster is closely related to the #1 Alzheimer’s disease cluster. Microglia play an important role in the pathological process of Alzheimer’s disease. Their abnormal activation can lead to neuroinflammation, which in turn promotes the deposition of *β*-amyloid and the hyperphosphorylation of tau protein, aggravating the condition of Alzheimer’s disease (Heppner et al., 2015; Maphis et al., 2015; Hong et al., 2016; Keren-Shaul et al., 2017; Sarlus and Heneka, 2017; Li Y. et al., 2023). The #7 gut microbiota cluster and the #9 immune response cluster interact through the “gut–brain axis.” The imbalance of gut microbiota can trigger a systemic inflammatory response, affect the balance of the immune system, and then affect the immune status of the central nervous system, which is closely related to the occurrence and development of cognitive impairment (Sampson et al., 2016; Harach et al., 2017; Li N. et al., 2023; Zha et al., 2025). The associations between these clusters highlight the importance of cross-topic research. Future research should focus on integrating the research content of different clusters and deeply exploring the complex network relationships between cognitive impairment and immunity.

The keyword timeline diagram indicates that microglia, neurodegeneration, gut microbiota, and the kynurenine pathway are current research hotspots. The Alzheimer’s disease cluster has been a research hotspot for decades. Over time, the research has deepened, gradually developing from a simple description of pathological features to the research on immune mechanisms and the exploration of immunotherapy strategies. It is expected that future research will continue to focus on precision medicine and personalized treatment. The emerging cluster, such as the #7 gut microbiota cluster, has rapidly gained attention in recent years, reflecting the importance attached to the interaction between gut microbiota–brain–immune system in the research field. The identification of TREM2 and gut microbiota as emerging research foci suggests promising directions for therapeutic intervention. Modulating the gut–immune–brain axis, for instance, has demonstrated potential in improving cognitive outcomes in both preclinical and early clinical studies. Future research is expected to make breakthroughs in revealing new pathogenesis and developing innovative treatment methods.

Analyzing highly cited literature facilitates the identification of research outcomes with significant impact and offers crucial insights for future research directions. The top 15 highly cited publications underscore the importance of foundational research and theoretical reviews in the field of immune and cognitive impairment. However, they also highlight the clinical translation of findings in immune and cognitive impairment is still in its early stages. Basic research has clearly indicated that neuroinflammation plays a pivotal role in the pathogenesis of Alzheimer’s disease (AD). Microglia, the resident immune cells in the central nervous system, become activated and undergo functional changes in AD, serving as primary contributors to neuroinflammation. Microglia exhibit diverse phenotypes under different conditions, exerting multifaceted influences on the progression of AD. The M1 pro-inflammatory phenotype, triggered by pathogen-associated molecular patterns (PAMPs) or damage-associated molecular patterns (DAMPs), releases neurotoxic substances, leading to neuronal damage and synaptic dysfunction, thereby accelerating the progression of AD. In contrast, the M2 anti-inflammatory/repair phenotype, triggered by anti-inflammatory factors (such as IL-4, IL-10, and TGF-*β*), promotes the clearance of Aβ, alleviates neuroinflammation, and protects neurons, thereby slowing the pathological progression of AD. Disease-associated microglia (DAM) is a unique phenotype of microglia that expresses specific genetic markers (such as TREM2 and APOE). DAM may play a protective role in the early stages of AD, but in the late stages of the disease, it may shift to a pro-inflammatory phenotype, exacerbating pathological damage. TREM2 mutations may increase the risk of AD by affecting the Aβ clearance capacity and inflammatory responses of microglia. Furthermore, other cells such as astrocytes and T cells interact with microglia through the release of inflammatory cytokines like IFN-*γ*, IL-1β, and TNF-*α*, collectively impacting the pathological process of AD (Heneka et al., 2015; Heppner et al., 2015; Liddelow et al., 2017; Gate et al., 2020). Variants of immune-related genes, such as TREM2, as well as the TREM2-APOE pathway, can drive transcriptional phenotypic changes in microglia, affecting their function and the pathogenesis of AD (Guerreiro et al., 2013; Krasemann et al., 2017; Ulrich et al., 2018; Parhizkar et al., 2019).

Neuroinflammation, gut microbiota, and genetic factors are currently prominent topics in cognition-related research. The analysis of keywords and co-citation clusters reveals evolving focal points and developmental trends in immune and cognitive impairment research. Cluster analysis of co-cited literature from 1985 to 2024 shows that the neuroinflammatory marker YKL-40, gut microbiota, and T cells form major clusters. For the co-cited literature cluster analysis from 2021 to 2024, dysbiosis of the gut ecosystem and probiotics, the gut–brain axis and therapy, inflammasomes and amyloidosis, and the emergence of microglia indicate that there has been increased focus on the specific states of gut dysbiosis and the use of probiotics to regulate gut microbiota, which may subsequently influence immunity and cognitive impairments. At the same time, these changes may play a significant role in immunity and cognitive impairments through the gut–brain axis. The co-occurrence of “inflammasome, ““amyloidosis,” and “microglia” indicates that the bidirectional role of microglia in cognition and inflammation is increasingly recognized. Inflammasomes, amyloidosis, and microglia interact and influence each other during the onset and progression of immune and cognitive disorders. The activation of inflammasomes can promote microglial activation and inflammatory responses, accelerating the process of amyloidosis; conversely, amyloidosis can activate inflammasomes and microglia, forming a vicious cycle that jointly drives the development of immune abnormalities and cognitive impairments. An in-depth study of their relationships helps to reveal the pathogenesis of immune and cognitive disorder-related diseases, providing a theoretical basis for the development of new therapeutic strategies. The relevance of metabolic syndrome, T cells, and TREM2 remains strong. TREM2, metabolic syndrome, YKL-40, LW-AFC, and lipopolysaccharide are all related to T cells, while YKL-40 and LW-AFC are also associated with gut microbiota. #2cluster T cells is also a major cluster generating a high number of citations. In view of current research trends, researchers should pay more attention to emerging hotspots. For example, in TREM2-related research, more studies should deeply analyze its signaling pathway and mechanism of action in neurodegenerative diseases such as Alzheimer’s disease, and explore the development of drugs targeting TREM2, including small molecule inhibitors or agonists, and TREM2-based immunomodulatory therapies, which are expected to provide new breakthroughs for the treatment of neurodegenerative diseases.

### 4.3 Strengths and limitations

Compared with the literature focusing on the immune mechanisms of Alzheimer’s disease, this study covered a wider range of cognitive impairment-related diseases, such as cognitive impairment caused by HIV infection and multiple sclerosis. The research perspective was more comprehensive and could analyze the immune commonalities and differences in cognitive impairment caused by different etiologies. However, in terms of the depth of research on the immune-related aspects of Alzheimer’s disease, it may not be as detailed as the research specifically targeting this disease. Additionally, regional disparities in citation impact should be acknowledged, which may stem from differences in language use, journal visibility, and international collaboration networks—particularly affecting institutions from non-English-speaking countries.

## Data Availability

The original contributions presented in the study are included in the article/Supplementary material, further inquiries can be directed to the corresponding authors.
