# Correction: A bibliometric analysis of the immune system and cognitive impairment: trends from 1985 to 2024

**DOI:** 10.3389/fnagi.2025.1675716

**Published:** 2025-08-22

**Authors:** Beibei Zou, Jinxi Xiang, Muhua Zhang, Jing Huang, Chao Feng

**Affiliations:** ^1^Department of Cardiology, The Fourth Affiliated Hospital of School of Medicine, International School of Medicine, International Institutes of Medicine, Zhejiang University, Yiwu, China; ^2^Shanghai Weiyu High School, Shanghai, China; ^3^Xuhui District Central Hospital, Shanghai, China

**Keywords:** cognitive impairment, immune system, bibliometric, neuroinflammation, microglia, gut microbiota, TREM2, co-citation

In the published article, there was a mistake in Table 4. In the original table, three specific data fields (not including the header row) were left unfilled: the country fields in columns 5 and 7, and the institute field in column 10, which caused some data misalignment in the published version. The corrected Table 4, with the missing fields completed and alignment issue resolved, appears below.

**Table 4:** Characteristics of the top 15 articles based on citations from 1985 to 2024.

**Rank**	**Title**	**Year**	**Citation**	**Total link strength**	**Author**	**Institute**	**Country**	**Journal**	**Type**
1	Neuroinflammation in Alzheimer's disease	2015	193	705	Heneka MT	University Hospital Bonn	Germany	Lancet Neurol	Review
2	From inflammation to sickness and depression: when the immune system subjugates the brain	2008	139	178	Robert Dantzer	University of Illinois	USA	Nat Rev Neurosci	Review
3	Inflammation and Alzheimer's disease	2000	132	449	Akiyama H	Sun Health Research Institute	USA	Neurobiol Aging	Review
4	Updated research nosology for HIV-associated neurocognitive disorders	2007	129	165	Antinori A	Istituto di Ricovero e Cura a Carattere Scientifico (IRCCS)	Italy	Neurology	Review
5	HIV-associated neurocognitive disorders persist in the era of potent antiretroviral therapy: CHARTER Study	2010	122	167	Heaton RK	University of California	USA	Neurology	Clinical research
6	NLRP3 is activated in Alzheimer's disease and contributes to pathology in APP/PS1 mice	2013	110	527	Heneka MT	University of Bonn	Germany	Nature	Research
7	Complement and microglia mediate early synapse loss in Alzheimer mouse models	2016	107	451	Hong S	Boston Children's Hospital and Harvard Medical School	USA	Science	Research
8	A Unique Microglia Type Associated with Restricting Development of Alzheimer's Disease	2017	103	470	Keren-Shaul H	Weizmann Institute of Science	Israel	Cell	Research
9	Neurotoxic reactive astrocytes are induced by activated microglia	2017	103	337	Liddelow SA	Stanford University	USA	Nature	Research
10	“Mini-mental state”. A practical method for grading the cognitive state of patients for the clinician	1975	101	168	Folstein MF	The New York Hospital-Cornell Medical Center	USA	J Psychiat Res	Research
11	TREM2 variants in Alzheimer's disease	2013	101	565	Guerreiro R	University College London (UCL) Institute of Neurology	UK	New Engl J Med	Meta
12	The amyloid hypothesis of Alzheimer's disease: progress and problems on the road to therapeutics	2022	99	379	Hardy J	National Institute on Aging	USA	Science	Review
13	The diagnosis of dementia due to Alzheimer's disease: recommendations from the National Institute on Aging-Alzheimer's Association workgroups on diagnostic guidelines for Alzheimer's disease	2011	96	225	Mckhann GM	Johns Hopkins University School of Medicine	USA	Alzheimers Dement	Comments
14	Immune attack: the role of inflammation in Alzheimer disease	2015	92	397	Heppner FL	Charité - Universitätsmedizin Berlin	Germany	Nat Rev Neurosci	Review
15	Immune modulation of learning, memory, neural plasticity and neurogenesis	2011	92	166	Yirmiya R	The Hebrew University of Jerusalem	Israel	Brain Behav Immun	Review

The original version of this article has been updated.

