# Supplementary material for: A bibliometric analysis of the immune system and cognitive impairment: trends from 1985 to 2024
Source: Front Aging Neurosci. 2025 Jul 28;17:1587575. doi: 10.3389/fnagi.2025.1587575 (PMC12336137; doi:10.3389/fnagi.2025.1587575)
Supplement: Supplementary file 1 [file Supplementary_file_1.docx]

**Supplementary Table S1: Characters of the top 20 authors based on publications from 1985 to 2024**

| rank | author | documents | citations | TLS | H_index | M_index | institute | country |
| --- | --- | --- | --- | --- | --- | --- | --- | --- |
| 1 | maes, michael | 29 | 1465 | 38 | 16 | 1 | University of Electronic Science and Technology of China | China |
| 2 | clerici, mario | 21 | 989 | 109 | 14 | 0.933 | University of Milano | Italy |
| 3 | barichello, tatiana | 17 | 608 | 55 | 12 | 0.923 | University of Texas Health Science Center at Houston | USA |
| 4 | fuchs, dietmar | 16 | 425 | 23 | 12 | 0.48 | Innsbruck Medical University | Austria |
| 5 | zetterberg, henrik | 16 | 358 | 24 | 12 | 1.33 | Göteborg University | Sweden |
| 6 | nemni, raffaello | 14 | 832 | 82 | 13 | 0.867 | University of Milano | Italy |
| 7 | ellis, ronald j. | 13 | 983 | 36 | 13 | 0.722 | University of California San Diego | USA |
| 8 | felipo, vicente | 13 | 211 | 23 | 8 | 13 | Centro de Investigación Príncipe Felipe | spain |
| 9 | grant, igor | 13 | 736 | 52 | 10 | 0.385 | University of California-San Diego | USA |
| 10 | larbi, anis | 13 | 482 | 42 | 10 | 13 | Nanyang Technological University | Republic of Singapore. |
| 11 | morgello, susan | 13 | 1086 | 28 | 13 | 0.929 | The Mount Sinai Medical Center | USA |
| 12 | teixeira, antonio lucio | 13 | 607 | 23 | 18 | 1.2 | The University of Texas Health Science Center at Houston | USA |
| 13 | agostini, simone | 12 | 245 | 63 | 10 | 12 | IRCCS Fondazione Don Carlo Gnocchi ONLUS | Italy |
| 14 | gendelman, he | 12 | 683 | 0 | 19 | 22 | University of Nebraska Medical Center | USA |
| 15 | heneka, michael t. | 12 | 1552 | 5 | 12 | 15 | German Center for Neurodegnerative Diseases | Germany |

**Supplementary Table S2: Characters of the top 15 authors based on publications from 2021 to 2024**

| rank | author | documents | citations | TLS | H_index | M_index | institute | country |
| --- | --- | --- | --- | --- | --- | --- | --- | --- |
| 1 | felipo, vicente | 11 | 80 | 36 | 6 | 8 | Centro de Investigación Príncipe Felipe | spain |
| 2 | maes, michael | 9 | 79 | 11 | 6 | 8 | University of Electronic Science and Technology of China | China |
| 3 | zetterberg, henrik | 8 | 45 | 7 | 5 | 6 | Göteborg University | Sweden |
| 4 | llansola, marta | 7 | 55 | 25 | 4 | 7 | Principe Felipe Research Center, Valencia | Spain |
| 5 | heneka, michael t. | 6 | 335 | 2 | 6 | 6 | German Center for Neurodegnerative Diseases | Germany |
| 6 | wang, xin | 6 | 64 | 1 | 4 | 6 | Huazhong University of Science and Technology | China |
| 7 | yu, jin-tai | 6 | 129 | 12 | 5 | 6 | Fudan university | China |
| 8 | cutuli, debora | 5 | 35 | 8 | 4 | 5 | University of Rome La Sapienza | Italy |
| 9 | decandia, davide | 5 | 35 | 8 | 4 | 5 | IRCCS Fondazione Santa Lucia | Italy |
| 10 | gaetani, lorenzo | 5 | 118 | 16 | 4 | 5 | University of Perugia | Italy |
| 11 | montoliu, carmina | 5 | 21 | 18 | 3 | 4 | University of Valencia | Spain |
| 12 | parnetti, lucilla | 5 | 118 | 16 | 4 | 5 | University of Perugia | Italy |
| 13 | ulvik, arve | 5 | 8 | 9 | 2 | 2 | Laboratoriebygget | Norway |
| 14 | wang, yao | 5 | 29 | 6 | 3 | 5 | Capital Medical University | China |
| 15 | almulla, abbas f. | 4 | 45 | 9 | 4 | 4 | Chulalongkorn University | Thailand |

**Supplementary Table S3: Characters of the top 8 clusters based on size from 1985 to 2024**

| Cluster | Size | Silhouette | mean(Year) | Top Term(log-likelihood ratio) | The top 3 co-cited authors |
| --- | --- | --- | --- | --- | --- |
| 0 | 217 | 0.942 | 2003 | HIV | HEATON RK（190），ANTINORI A（120），SACKTOR N（94） |
| 1 | 157 | 0.888 | 2010 | hippocampus | DANTZER R（227），ZHANG Y（219），PERRY VH（190） |
| 2 | 155 | 0.822 | 2010 | alzheimers disease | MCGEER PL（201），AKIYAMA H（150），HOLMES C（142） |
| 3 | 145 | 0.823 | 2016 | microglia | RANSOHOFF RM（163），LIU Y（162），WANG J（157） |
| 4 | 90 | 0.913 | 2020 | nlrp3 inflammasome | HENEKA MT（383），WANG Y（140），HEPPNER FL（91） |
| 5 | 88 | 0.875 | 2019 | t cells | BARUCH K（113），SARESELLA M（100），IADECOLA C（87） |
| 6 | 87 | 0.933 | 2014 | schizophrenia | MEYER U（143），BROWN AS（136），CHEN Y（52） |
| 7 | 80 | 0.938 | 2020 | gut microbiota | ZHANG J（98），ZHANG L（92），CRYAN JF（88） |

**Supplementary Table S4: Characters of the top 8 clusters based on size from 2021 to 2024**

| Cluster | Size | Silhouette | mean(Year) | Top Term(log-likelihood ratio) | The top 3 co-cited authors |
| --- | --- | --- | --- | --- | --- |
| 0 | 65 | 0.923 | 2021 | gut microbiota | CRYAN JF（74），ZHANG L（71），ERNY D（51） |
| 1 | 54 | 0.846 | 2021 | trem2 | KEREN-SHAUL H（75），KRASEMANN S（51），GUERREIRO R（50） |
| 2 | 53 | 0.874 | 2022 | t cells | GATE D（62），SWEENEY MD（62），BARUCH K（61） |
| 3 | 42 | 0.936 | 2022 | nlrp3 inflammasome | WANG Y（109），LIU Y（91），WANG H（60） |
| 4 | 40 | 0.872 | 2022 | depression | DANTZER R（70），LI J（65），LIU Q（53） |
| 5 | 36 | 0.868 | 2021 | alzheimers disease | JACK CR（77），SELKOE DJ（58），HAMPEL H（43） |
| 6 | 31 | 0.881 | 2021 | synapse loss | HENEKA MT（193），HONG S（82），PAOLICELLI RC（70） |
| 7 | 30 | 0.877 | 2021 | frailty | LI Y（82），LIDDELOW SA（81），YANG Y（75） |
